# The power of poop: Defecation behaviors and social hygiene in insects

**DOI:** 10.1371/journal.ppat.1009964

**Published:** 2021-10-28

**Authors:** Marissa E. Cole, Javier A. Ceja-Navarro, Aram Mikaelyan

**Affiliations:** 1 Department of Entomology and Plant Pathology, North Carolina State University, Raleigh, North Carolina, United States of America; 2 Joint BioEnergy Institute, Emeryville, California, United States of America; 3 Bioengineering and Biomedical Sciences Department, Biological Systems and Engineering Division, Lawrence Berkeley National Laboratory, Berkeley, California, United States of America; 4 Institute for Biodiversity Science and Sustainability, California Academy of Sciences, San Francisco, California, United States of America; University of Maryland, Baltimore, UNITED STATES

Feeding and defecation are necessary biological processes. Although the main purpose of defecation is waste elimination, it has also had a profound influence on the evolution of animal behavior and ecological interactions. Due to the capacity of fecal material to support the growth of microbes, including potential pathogens, defecation has direct consequences on animal health. To counter these risks, insects have evolved a range of unique behavioral and physiological adaptations, often involving microbial symbionts; these adaptations are particularly important for social insects, which are at an especially increased risk of fecal exposure and associated disease due to more crowded living conditions and high site fidelity.

## Insects practice “fecal” distancing

**Fig 1:**
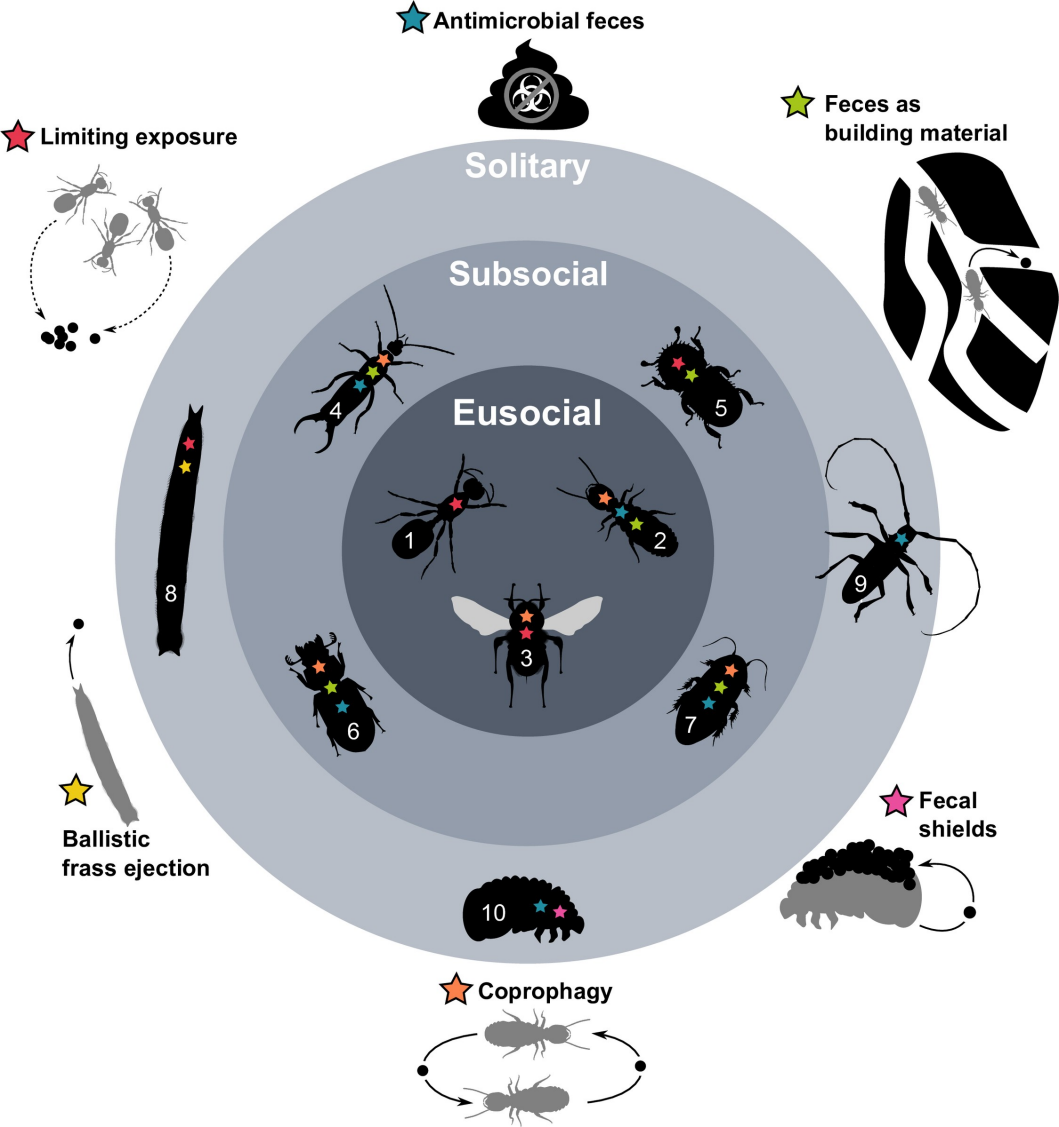
A summary of the diversity of defecation behaviors found among eusocial [ants (1), termites (2), and bees (3)], subsocial [earwigs (4), bark and ambrosia beetles (5), passalid beetles (6), and wood roaches (7)], and solitary insects [caterpillars (8), longhorn beetles (9), and leaf beetle larvae (10)]. Not all the behaviors are necessarily found in a given species within each group. Most insect species minimize pathogen risk by reducing their exposure to feces, but several social insects that are forced to live in close proximity to feces instead do so by minimizing the pathogen growth in feces. The presence of antimicrobial compounds in insect feces appears to have enabled the evolution of behaviors in insects that require an intimate contact with feces, such as its use in nest building, the use of fecal shields to avert predators.

Because feces is rich in organic matter, it can be an effective disease reservoir [1]. The risk of feces-related disease is more pronounced in species with high site fidelity, living in close quarters with each other and/or their feces [1]. Honey bees, for example, go for “cleansing flights” (Fig 1) and defecate exclusively outside of the hive [2]. Several species of caterpillars, which live in stationary leaf shelters, have also evolved behaviors such as “ballistic frass ejection,” shooting fecal pellets (Fig 1) to remarkable distances, successfully avoiding accumulation of fecal material and subsequent need for removal [3]. While feces avoidance mostly seems to benefit invertebrates by preventing disease, it is not the only benefit—predatory and parasitic wasps seem to have the ability to use volatile chemicals from frass to locate their host species [4,5], and some lepidopteran and coleopteran species have shown oviposition avoidance in areas that have been treated with feces-associated volatiles of their own species and that of others, potentially saving their offspring from future competition [6,7].

With the evolution of subsociality and eusociality came an increased exposure to fecal material due to living in large groups of individuals in a localized area. In response to increased likelihood of contracting pathogens, a wide range of hygienic behaviors evolved, which are specific to social insects: “social immunity,” a set of behavioral adaptations enabling insects to collectively prevent infection, including waste removal, grooming, cannibalism, undertaking, and maintaining “latrines” outside of nests [1,8]. For instance, *Myrmica rubra* worker ants, despite an increased risk of exposure to pathogens, remove more waste upon detection of pathogenic fungal conidia on feces when brood are present [9].

## Sociality and beneficial relationships with feces

Although it may seem paradoxical, several behaviors in insects have simultaneously evolved keeping individuals closer to fecal material. Pyralid moth larvae [10], some bark beetles [11,12], and termites [13] use frass to build their dwellings (Fig 1). Dark garden ants maintain fecal patches around the nest referred to as “kitchen middens,” which are not cleared out with other waste and are presumably treated by ants to limit microbial growth [14]. Additionally, ambrosia beetles, known for farming “ambrosia fungi,” may be provided essential elements by their obligate fungal symbiont through the recycling of fecal material (Fig 1), with the highest concentrations of essential elements found to be positively correlated with the beetle’s level of sociality [15]. This correlation suggests that ambrosia beetles may benefit nutritionally from being more social and in closer association with a higher volume of feces.

Some leaf beetles in the family Chrysomelidae produce “fecal shields,” which serve as physical protection but are also fortified with plant-derived metabolites such as steroidal alkaloids, saponins, an array of fatty acids, and phytol, which have been suggested to play a role in deterring predators [16]. Although chrysomelid fecal shields serve as physical and chemical barriers against predators (Fig 1), many of the aforementioned metabolites also have demonstrable antimicrobial properties [2]. Regardless of the degree of contact, it is reasonable to assume that all behaviors involving close contact with feces must have evolved alongside mechanisms to regulate pathogen growth, which may rely on components derived from the diet or gut microbiome.

## Protected by poop: The use of feces for nest sanitation and gut health

Evidence for antimicrobial benefits of feces comes primarily from social insects, first demonstrated in the termite *Zootermopsis angusticollis*, where fecal matter was shown to have a dose-dependent inhibitory effect on spore germination in the entomopathogenic fungus *Metarhizium anisopliae* [17]. In addition, protozoans in the gut of *Z*. *angusticollis* synthesize enzymes targeting the predominant polysaccharide in the fungal cell wall, indicating potential for digestion of fungi and/or protection from fungal pathogens [18]. A similar effect was later observed with feces from the subsocial wood roach *Cryptocercus punctulatus* (Fig 1), where it was proposed that the active antifungal components were likely of microbial origin [19]. Given that termites and wood roaches are sister clades, this apparent defensive role of the fecal microbiome likely emerged in their common ancestor. Antimicrobial properties have also been discovered in earwig excrement [20] and passalid beetles (Fig 1) [21], indicating that taxonomically widespread lineages of social insects have evolved similar strategies to inhibit pathogen growth. Antimicrobial effects of fecal microbiomes in social insects could be attributed to microbes colonizing the diet, surviving gut passage, and producing secondary metabolites in feces or those colonizing and selectively growing in feces.

Many social insects commonly engage in trophallaxis (Fig 1; exchange of gut fluids between individuals) and coprophagy (consumption of feces), which may allow sharing of microbiomes and metabolites among nestmates. Although these behaviors primarily serve nutritional roles, the potential antimicrobial role of feces in nest hygiene has been suggested to be an additional incentive for their selection in social insects [20]. Moreover, although mechanisms of pathogen inhibition by gut microbiomes are different from that of fecal microbiomes, gut communities have been suggested to play roles in preventing germination of fungal spores in guts of locusts [22] and termites [23]. It is therefore possible that coprophagy (Fig 1) evolved to “treat” feces by passing it through the intestinal tract to neutralize potential pathogens. Inoculation of individuals with fecal microbiome members could also have a function in preventing disease. In bumblebees, for example, the consumption of feces from healthy individuals has been shown to reduce parasite loads of *Crithidia bombi* [24,25]. Coprophagy has been suggested to play a similar role in social immunity of the European earwig [20], although the exact microbiome contributions to antimicrobial properties of its feces remain unclear and need more investigation.

## Weaponized poop: Antimicrobial potential of feces

Multiple insect species rely on wood as a source of food and shelter, although this means living in close quarters with nestmates and other species inside damp galleries within decaying logs. The conditions within decaying wood potentially amplify the risk of pathogen breakouts and impose selective pressures for evolution of the fecal microbiome’s role in social immunity. The microbiological and chemical bases of feces-mediated defenses have been best dissected for actinobacteria in the subsocial wood-feeding beetle, *Odontotaenius disjunctus*, with similar roles suggested for strains isolated from guts of fungus-cultivating termites [21,26]. Feces-associated Actinomycetes collected from various populations of the beetle across the United States were observed to collectively produce a rich cocktail of antimicrobial metabolites from multiple families (Fig 1) [21]. In vitro inhibition assays with the isolated actinobacterial strains demonstrated a strong ability to inhibit *M*. *anisopliae* [21].

Gut and fecal microbiomes of wood-feeding larvae of the longhorn beetle *Cerambyx welensii* harbor multiple actinobacterial strains producing a wide range of antimicrobial compounds (Fig 1), including several with broad ranges of bioactivities against potential pathogens [27]. Antifungal properties of fecal isolates from the herbivorous rhinoceros beetle *Allomyrina dichotoma* [28] and bark beetle *Dendroctonus rufipennis* (Fig 1) [29] suggest that actinobacteria might play a wider role in reducing pathogen load in gut microbiomes of lignocellulose-feeding beetles. Finally, in the Eastern subterranean termite, *Reticulitermes flavipes*, actinobacteria have been found in feces-lined galleries, surrounding soils, and on the termite cuticle; considering the occurrence of grooming and coprophagy in termites, it is possible that actinobacteria are cycled throughout the colonies and nests themselves [30]. The subterranean termite pest, *Coptotermes formosanus*, may also be provided with disease resistance by actinobacteria associated with their fecal comb nests [31]. Incidentally, multiple species of fungus-feeding termites have also been found to be associated with actinobacterial strains encoding a wide array of novel biosynthetic gene clusters as well as putative chitinases postulated to play a protective role against microbial pathogens [26,32].

## Future work

Social immunity is a burgeoning field; we are only beginning to understand ecological roles and microbial bases of some hygienic defecation behaviors in insects. Studying the composition and functional potential of fecal microbiomes, especially from wood-feeding insects, holds considerable promise to not only better our understanding of the biology of insects, but also to the isolation of microbial strains with novel bioactive capabilities. Future work should clarify the extent to which diet and sociality of insect lineages play a role in determining the antimicrobial properties of feces and explore the ecological and evolutionary patterns among not just intestinal, but fecal microbiomes as well.
